# Supplementary material for: Blood meal acquisition enhances arbovirus replication in mosquitoes through activation of the GABAergic system
Source: Nat Commun. 2017 Nov 2;8:1262. doi: 10.1038/s41467-017-01244-6 (PMC5665997; doi:10.1038/s41467-017-01244-6)
Supplement: Supplementary file 1 — Supplementary Information [file 41467_2017_1244_MOESM1_ESM.pdf]

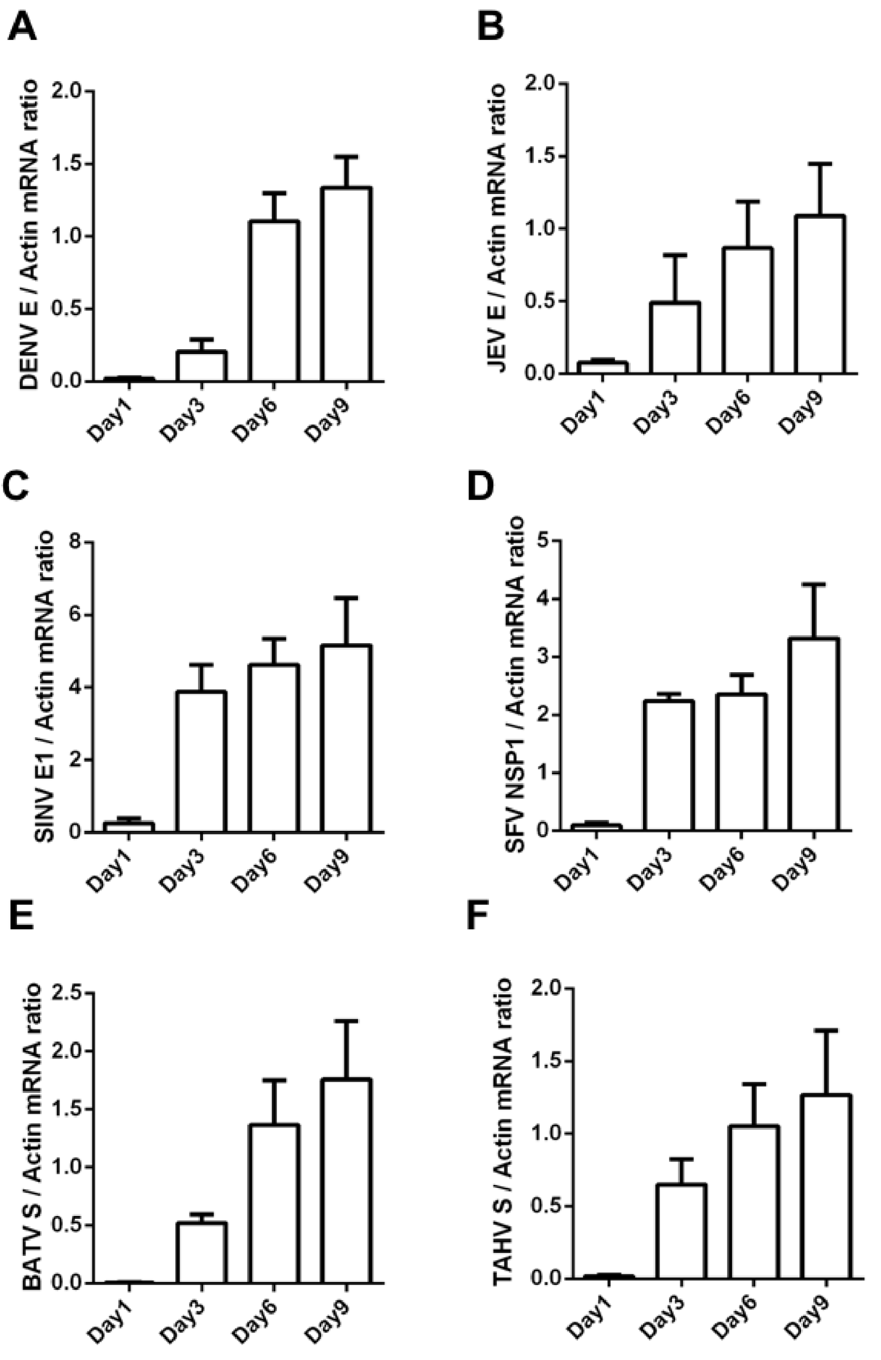

Supplementary Figure 1. The replication rates of different arboviruses in *A. aegypti*.

We used 100 M.I.D.$_{50}$ of DENV-2 (A), JEV (B), SINV (C), SFV (D), BATV (E) or TAHV (F) to infect the female *A. aegypti* mosquitoes by thoracic microinjection, respectively. The viral burdens in the whole mosquitoes were quantified by qPCR over a time course post-infection. The primers and probes for PCR detection are presented in Supplementary Data 2. There were at least five mosquitoes in each group. The data are presented as the mean ± s.e.m.

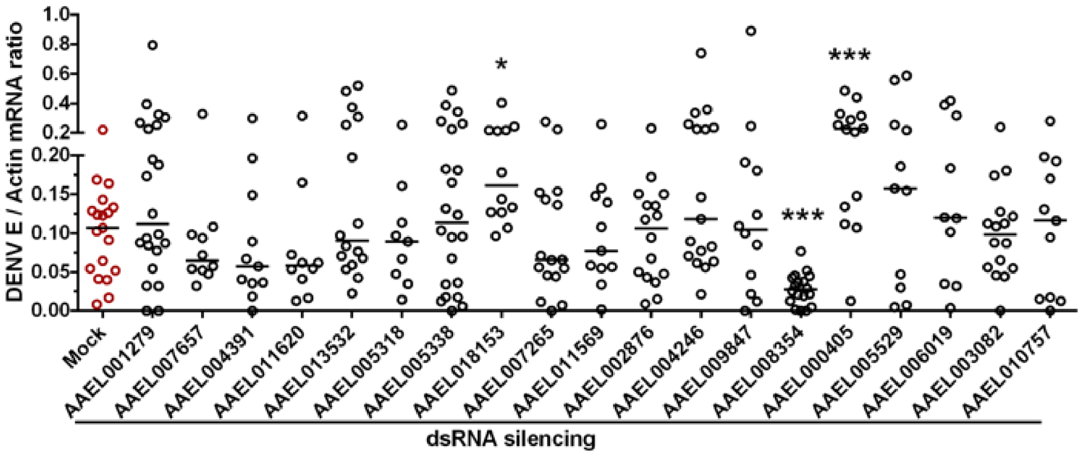

Supplementary Figure 2. The role of 19 arbovirus-induced genes in the DENV-2 infection of *A. aegypti*

Nineteen induced genes, which were consistently identified 6 days post-infection with arboviruses (Figure 1C), were individually silenced by dsRNA thoracic inoculation. *GFP* dsRNA was used as the mock control. Three days post-dsRNA treatment, 10 M.I.D.$_{50}$ of DENV-2 was microinjected into the dsRNA-treated mosquitoes. The viral loads were assessed on 3 days post-infection by TaqMan qPCR and were normalized to *A. aegypti actin* (*AAEL011197*). One dot represents 1 mosquito, and the horizontal line represents the median of the results. Differences were considered significant if $P < 0.05$. $*P < 0.05$; $**P < 0.01$; and $***P < 0.001$. The data were analyzed statistically using the non-parametric Mann-Whitney test. The results were combined from 2 independent experiments. The primers and probes for PCR and dsRNA synthesis are presented in Supplementary Data 2.

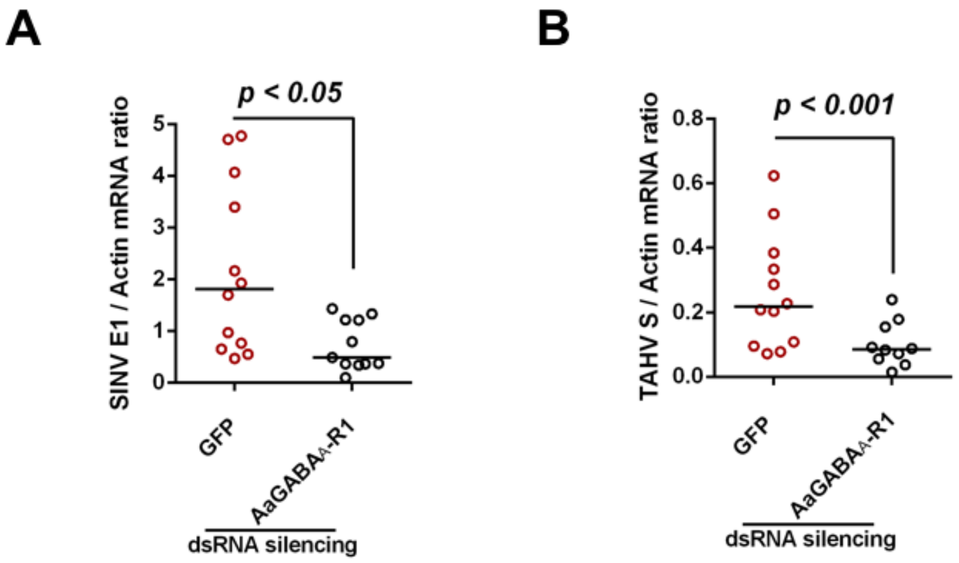

Supplementary Figure 3. Genetic impairment of *AaGABA$_A$-R1* reduced SINV and TAHV infections in *A. aegypti* mosquitoes

AaGABA$_A$-R1 was silenced by dsRNA inoculation. Mosquitoes inoculated with *GFP* dsRNA served as negative controls. Either 10 M.I.D.$_{50}$ of SINV (A) or TAHV (B) were inoculated into the dsRNA-treated mosquitoes at 3 days post dsRNA inoculation. The viral loads were determined 3 days post-infection. One dot represents 1 mosquito, and the horizontal line represents the median of the results. The data were analyzed statistically using the non-parametric Mann-Whitney test. The qPCR primers used are presented in Supplementary Data 2.

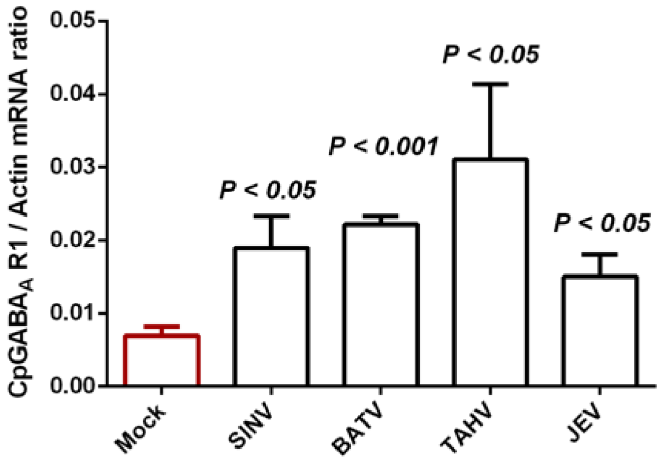

Supplementary Figure 4. Regulation of *CpGABA$_A$-R1* gene in *C. pipiens pipiens* infected by JEV, SINV, TAHV and BATV

100 MID$_{50}$ virus was thoracically microinjected a *Culex* mosquito. Mosquitoes inoculated by PBS served as a negative control. The *CpGABA$_A$-R1* abundance was assessed by SYBR Green qPCR on 6 days post-infection and was normalized by *Culex actin* (*CPIJ005786*). Each group included no less than eight *Culex* mosquitoes. The data were analyzed statistically by the non-parametric Mann-Whitney test. The data are presented as the mean ± s.e.m.

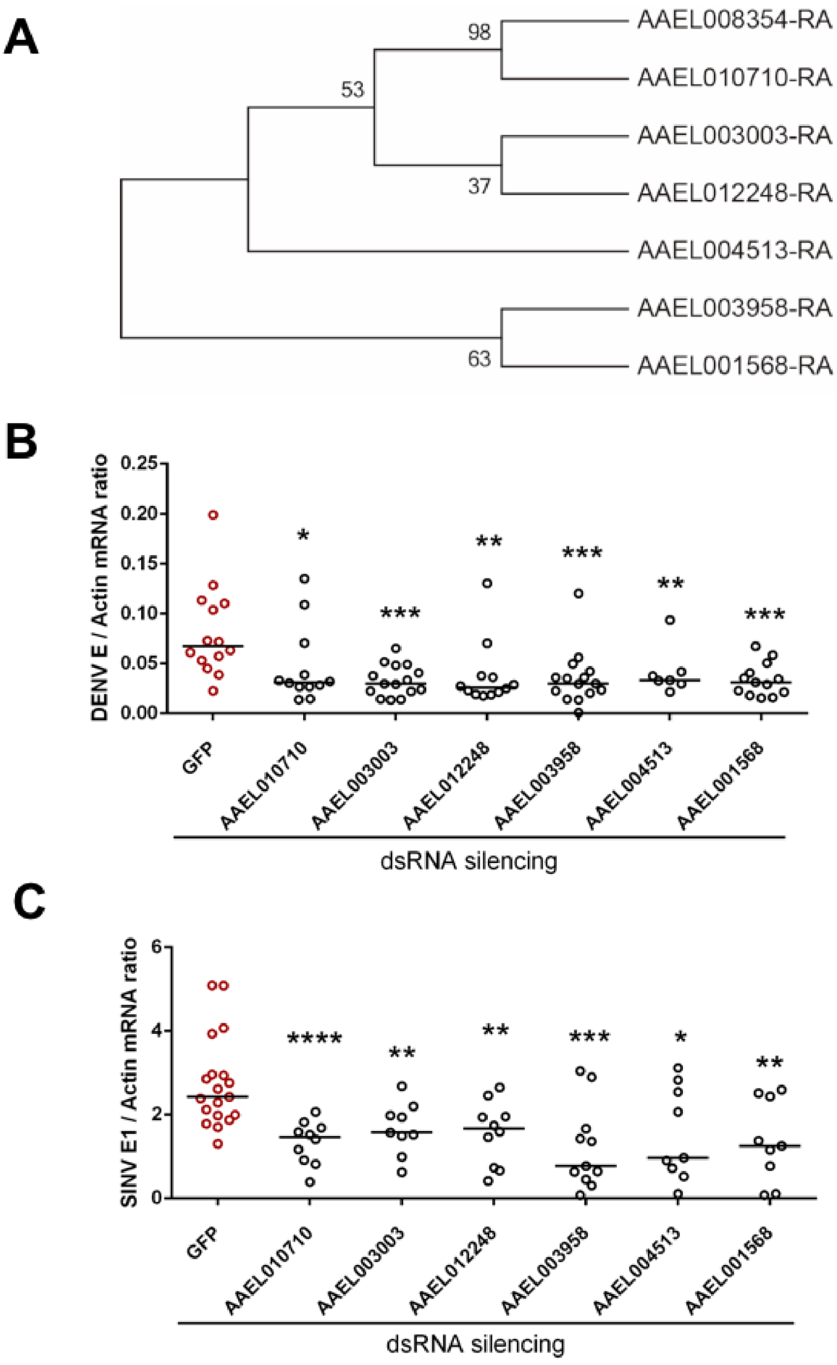

Supplementary Figure 5. The role of *AaGABA$_A$-R* genes in the arboviral infection of *A. aegypti*

(A) Unrooted phylogenetic tree of *AaGABA$_A$-R* genes. The tree was constructed using the neighbor-joining (NJ) method based on the alignment of these *AaGABA$_A$-R1* sequences.

(B-C) The role of *AaGABA$_A$-R* genes in the infection of *A. aegypti* by DENV-2 (B) and SINV (C). The *AaGABA$_A$-R* genes were individually silenced by thoracic inoculation with dsRNA.

GFP dsRNA was used as a mock control. Either 10 M.I.D.$_{50}$ of DENV-2 (B) or SINV (C) was microinjected into the dsRNA-treated mosquitoes 3 days post-dsRNA treatment. The viral loads were assessed on 3 days post-infection via Taqman qPCR or SYBR Green qPCR and were normalized to *A. aegypti actin* (*AAEL011197*). One dot represents 1 mosquito, and the horizontal line represents the median of the results. Differences were considered significant if $P < 0.05$. *$P < 0.05$; **$P < 0.01$; and ***$P < 0.001$. The data were analyzed statistically using the non-parametric Mann-Whitney test. The results from 2 independent experiments were combined. The primers and probes for PCR and dsRNA synthesis are presented in Supplementary Data 2.

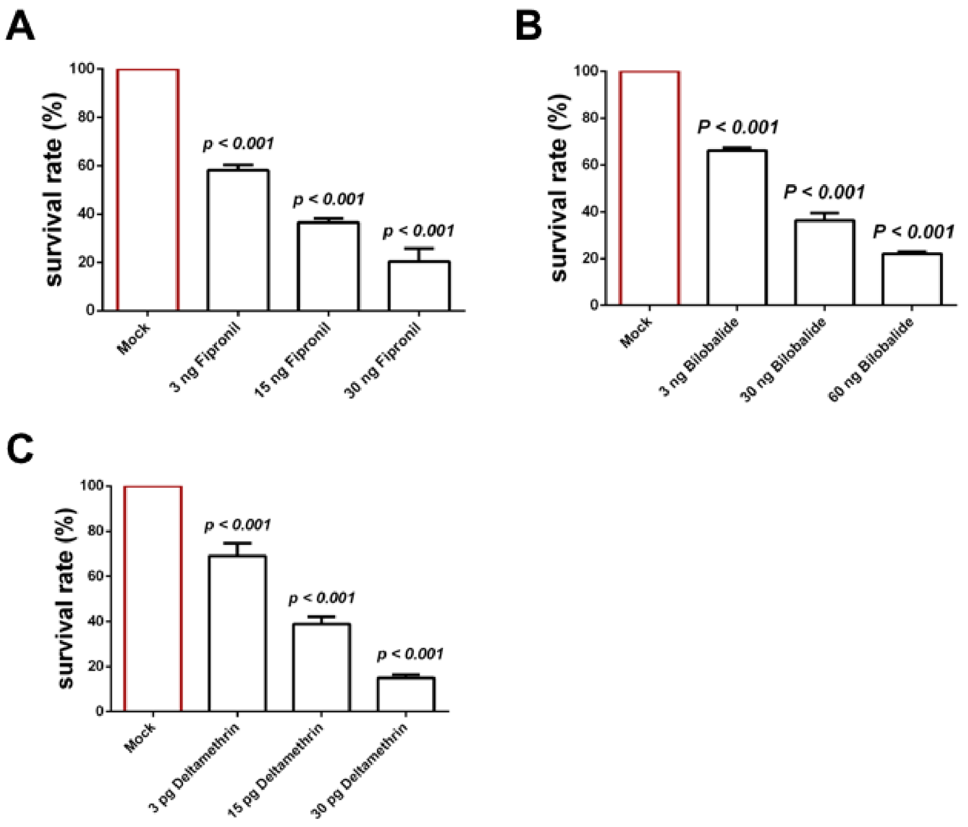

Supplementary Figure 6. Survival ratios of mosquitoes thoracically inoculated with insecticides

Serial concentrations of Fipronil (A), Bilobalide (B) and Deltamethrin (C) were microinjected into the thorax of *A. aegypti*. The survival rate was determined at 2 hours post-inoculation. The data were analyzed statistically using the Student's *t* test. The experiment was reproduced by 3 times. The data are presented as the mean ± standard error. The number on the X-axis represent the amount of insecticides inoculated per mosquitoes.

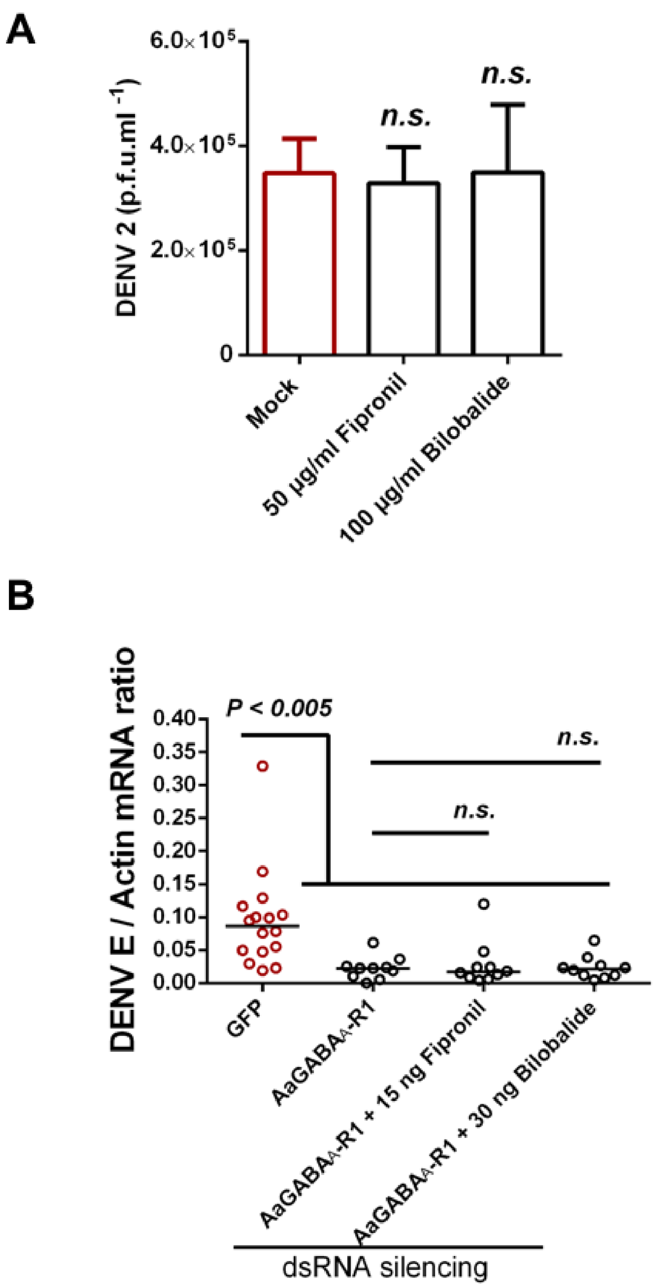

Supplementary Figure 7. The insecticides did not have additional effects on DENV-2 replication in the *AaGABA$_A$-R1* silencing mosquitoes.

(A) Incubation of Fipronil and Bilobalide did not affect the DENV-2 infectivity. 6 × 10^5 pfu DENV-2 was premixed with either 50 μg/ml Fipronil or 100 μg/ml Bilobalide for 30 mins, respectively. The virus incubated with PBS served as negative control. After 30 mins post-incubation, the viral titer was determined by a plaque assay. The experiment was reproduced by 2 times. The data are presented as the mean ± s.e.m.

B) Both GABA$_A$-receptor targeting insecticides did not have additional effects on DENV-2

replication in the *AaGABA$_A$-R1*-silenced mosquitoes. Either 15 ng of Fipronil or 30 ng of Bilobalide mixed with 10 M.I.D.$_{50}$ of DENV-2 were inoculated into the *AaGABA$_A$-R1* dsRNA-treated mosquitoes. The viral loads were assessed on 3 days post-infection via TaqMan qPCR and were normalized to *A. aegypti actin* (*AAEL011197*). The primers and probes used for PCR are presented in Supplementary Data 2. One dot represents 1 mosquito, and the horizontal line represents the median of the results. The data were analyzed statistically using the non-parametric Mann-Whitney test. The data from two independent experiments were combined. The number on the X-axis represent the amount of insecticides inoculated per mosquitoes.

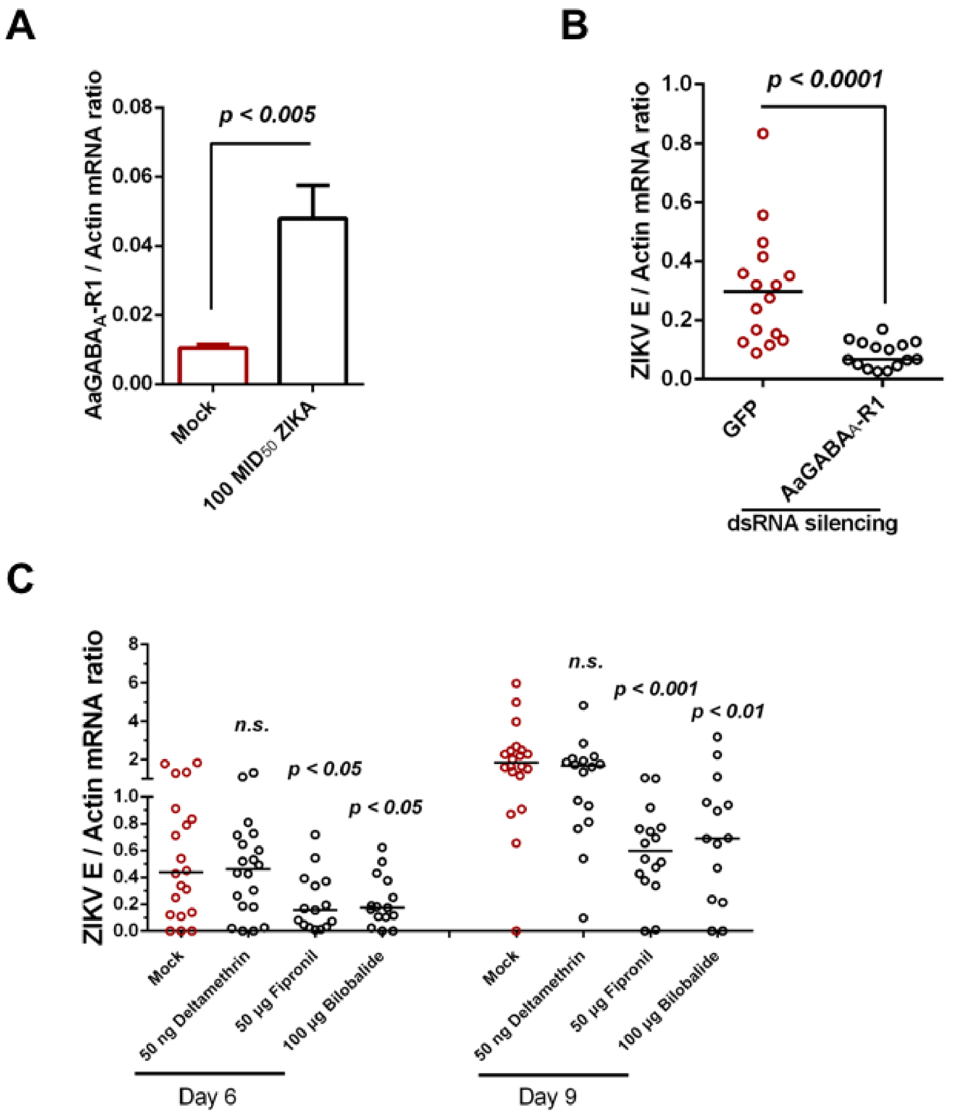

Supplementary Figure 8. The role of GABA$_A$ receptor-mediated GABA signaling in ZIKV infection of *A. aegypti*

(A) Induction of *AaGABA$_A$-R1* by ZIKV infection in *A. aegypti*. Ten M.I.D.$_{50}$ of ZIKV were inoculated into the thoracic region of mosquitoes. Mosquitoes that were microinjected with PBS served as mock controls. The abundance of *AaGABA$_A$-R1* was assessed by SYBR Green qPCR on 3 days post-infection and were normalized to *A. aegypti actin* (*AAEL011197*). The experiment was reproduced twice. The data are presented as the mean ± s.e.m.

(B) Knockdown of the *AaGABA$_A$-R1* gene reduced ZIKV replication in mosquitoes. Mosquitoes inoculated with *GFP* dsRNA served as negative controls. Ten M.I.D.$_{50}$ of ZIKV were inoculated on 3 days post-dsRNA inoculation. The viral loads were assessed on 3 days

post-infection by SYBR Green qPCR and were normalized to *A. aegypti actin* (*AAEL011197*).

(C) Insecticide exposure reduced ZIKV replication in *A. aegypti*. Mosquitoes infected by oral membrane blood feeding were exposed to bottles sprayed with Fipronil (50 μg/ bottle), Bilobalide (100 μg/ bottle) or Deltamethrin (50 ng/ bottle) for 2 hours. For mosquito oral infection, 5×10^5 p.f.u. ml^{-1} of ZIKV was used. Infected mosquitoes exposed to PBS served as mock controls. The mosquitoes that survived were transferred to new culture containers for further rearing. The viral loads were assessed over time post-infection by SYBR Green qPCR and were normalized to *A. aegypti actin* (*AAEL011197*).

(A-C) The data were analyzed statistically using the non-parametric Mann-Whitney test. The data from 2 independent experiments were combined.

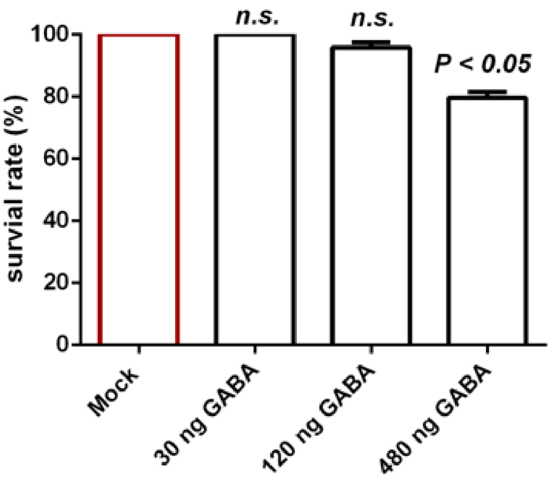

Supplementary Figure 9. Survival ratios of mosquitoes thoracically inoculated with GABA

A serial concentration of GABA were microinjected into the thorax of *A. aegypti*. The survival rate was determined at 2 hours post-inoculation. The data were analyzed statistically using the Student's *t* test. The experiment was reproduced three times. The data are presented as the mean ± standard error.

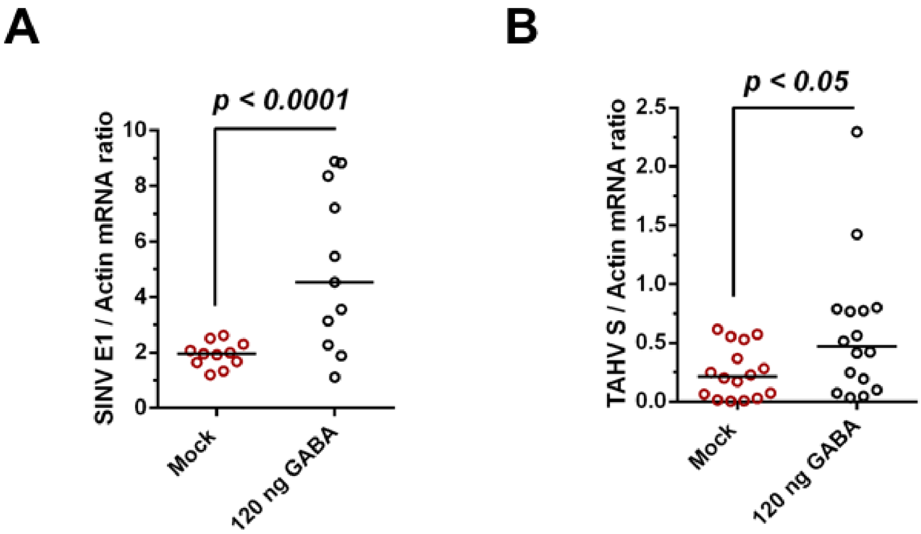

Supplementary Figure 10. Inoculation of GABA facilitated the SINV and TAHV infections in *A. aegypti*

Either 10 M.I.D.$_{50}$ of SINV (A) or TAHV (B) with 120ng of GABA were microinjected into the mosquito thoraxes. Mosquitoes inoculated with PBS and the viruses were used as mock controls. The viral loads were assessed on 3 days post-infection via SYBR Green qPCR and were normalized to *A. aegypti actin* (*AAEL011197*). One dot represents 1 mosquito, and the horizontal line represents the median of the results. The data were analyzed statistically using the non-parametric Mann-Whitney test. The results were combined by 2 independent experiments.

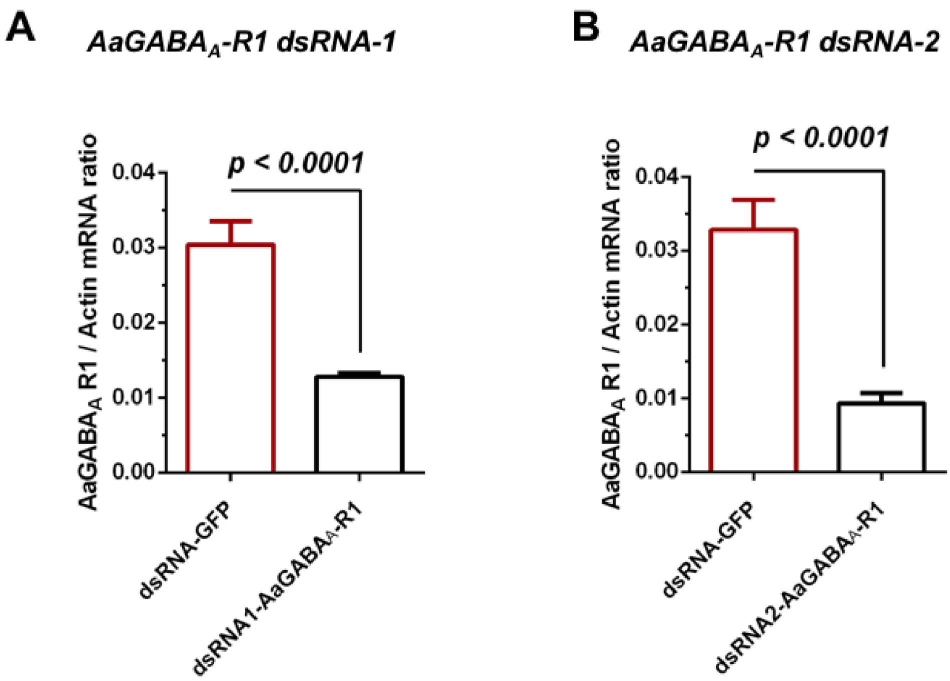

Supplementary Figure 11. Silencing efficiency of *AaGABA$_A$-R1* by dsRNA inoculation in *A. aegypti*

Two independent dsRNAs against the *AaGABA$_A$-R1* gene were inoculated into mosquitoes. Mosquitoes inoculated with *GFP* dsRNA served as negative controls. The abundance of *AaGABA$_A$-R1* was assessed by SYBR Green qPCR on 3 days post dsRNA inoculation. The data were analyzed statistically using the non-parametric Mann-Whitney test. The experiment was reproduced twice. The data are presented as the mean ± s.e.m.

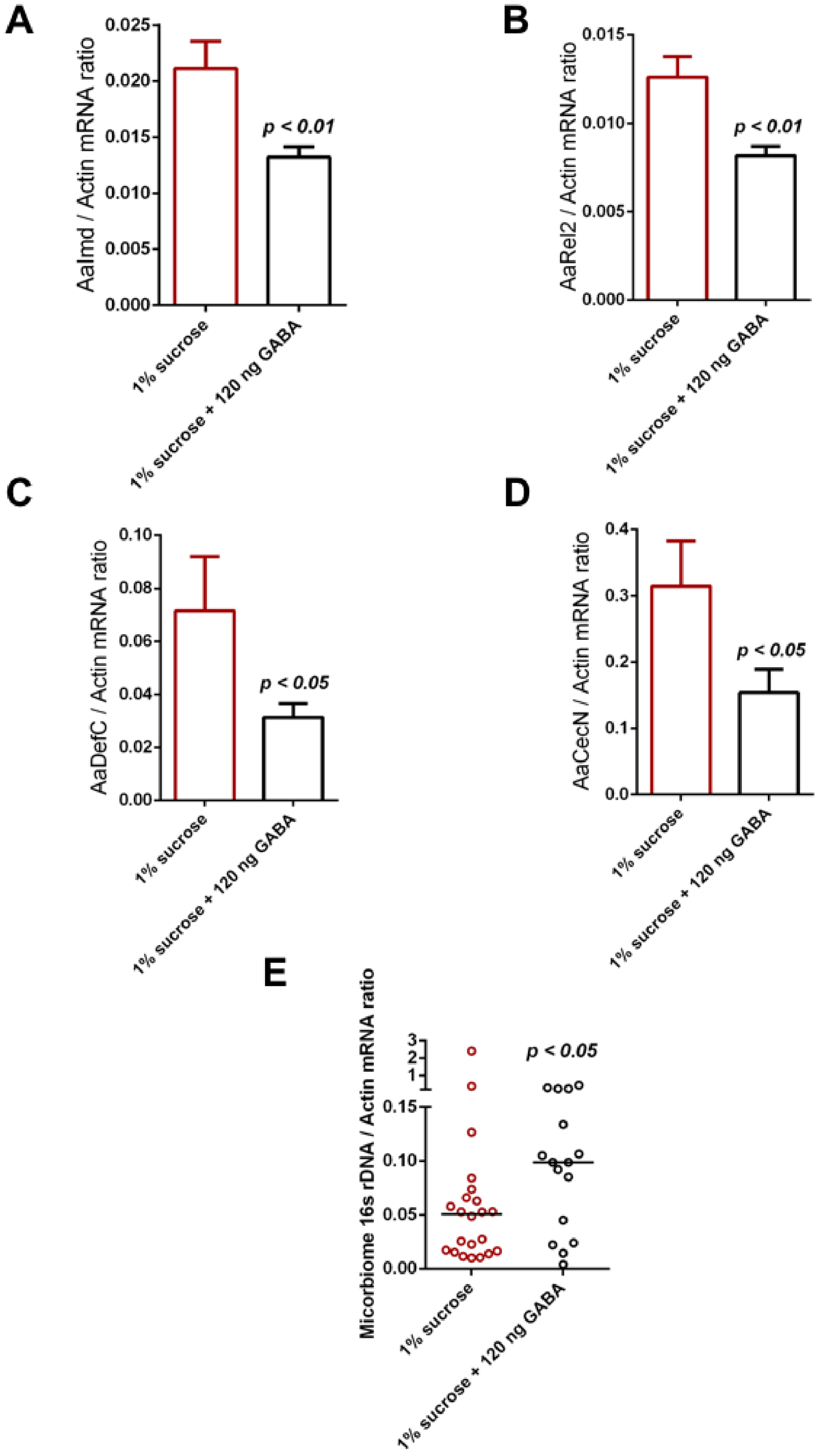

Supplementary Figure 12. Regulation of the Imd-mediated gut immunity by feeding with GABA.

(A-D) Regulation of Imd signaling by feeding with GABA. Since proliferation of the microbiota could skew the Imd signaling, the gut commensal microbiome was removed by

feeding antibiotics. The antibiotic-treated mosquitoes were fed by 120 ng of GABA with a sucrose meal. Antibiotic-treated mosquitoes fed a sucrose meal without GABA served as negative controls. The midguts were dissected at 18 hours after feeding to determine the expression of the *AaImd* (A), *AaRel2* (B), *AaDef-C* (C) and *AaCec-N* (D) genes by SYBR Green qPCR. The experiment was reproduced twice. The data are presented as the mean ± s.e.m.

(E) Regulation of the gut microbiome by feeding with GABA. Normal mosquitoes were fed with a sucrose meal, with or without 120ng of GABA. The midguts were dissected at 24 hours after feeding**.** The burden of the gut bacteria was determined by qPCR. The qPCR primers for 16S rDNA from the gut bacteria are described in Supplementary Data 2. One dot represents one mosquito gut. The horizontal line represents the median value of the results.

(A-E) The data were analyzed statistically using the non-parametric Mann-Whitney test. The qPCR primers for the *AaImd*, *AaRel2*, *AaDef-C, AaCec-N* genes and gut bacterial 16S rDNA are described in Supplementary Data 2.

A

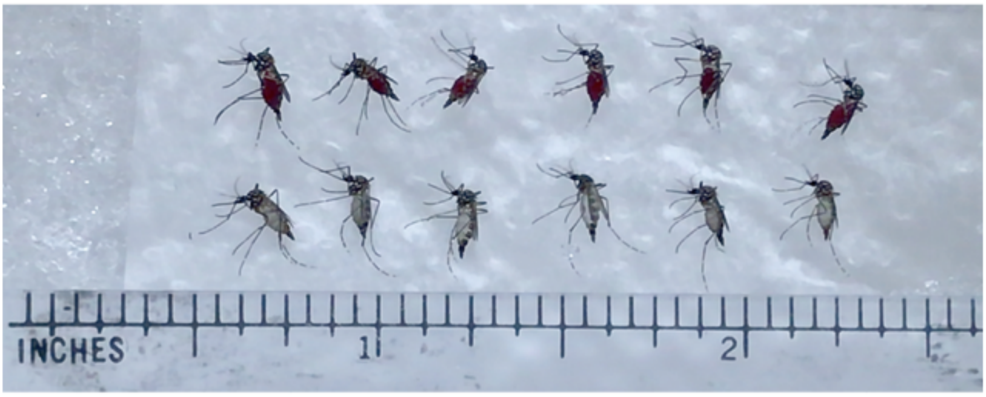

B

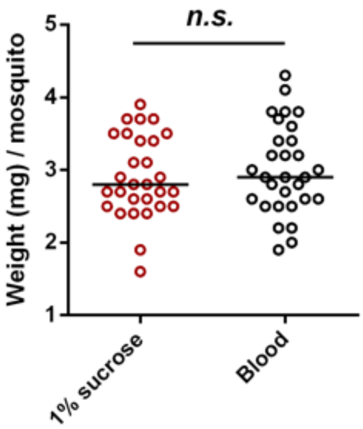

Supplementary Figure 13. Measurement of fully engorged mosquitoes immediately collected after either sucrose meal or blood feeding

The 5-7 days old female mosquitoes were fed by either 1% sucrose or the whole human blood, via feeders of the Hemotek feeding system. The fully engorged mosquitoes were immediately collected for the size (A) and weight (B) comparison. There was no significant difference in the size and weight of mosquitoes fed by sucrose and human blood. (B) One dot represents a mosquito. The data were statistically analyzed by the Student's *t* test.

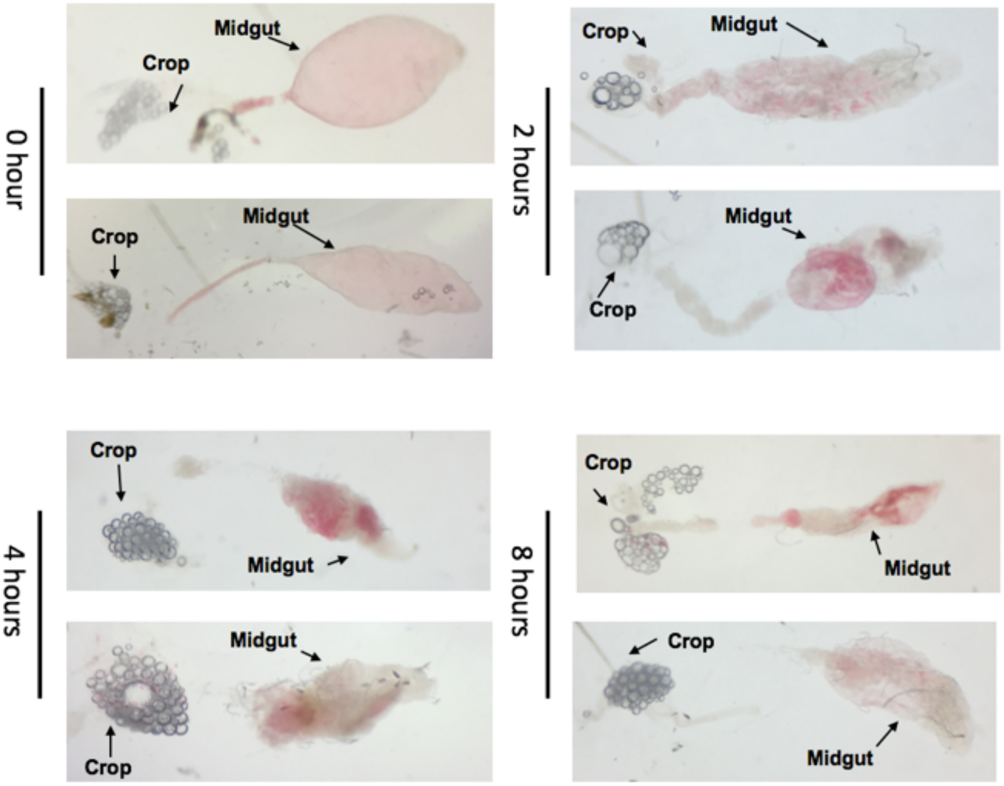

Supplementary Figure 14. Tracking the acquisition of sucrose liquid in the mosquito midgut and crop

The previous studies showed that digestion of sugar in insects may take place in either the midgut[40-42] or the sack-like crop[42]. The acquisition and digestion location is influenced by the meal size consumed, sugar concentration and other factors[43]. We therefore investigated which tissue is exactly used for stocking the virus-spiked sucrose liquid after a meal. The *A. aegypti* mosquitoes were fed by 1% sucrose (50% v/v), the supernatant from DENV-2-infected Vero cells (50% v/v), with 0.1% Ponceau S (the final concentration). Both the midgut and the crop were dissected at 0, 2, 4, 8 hours after the sucrose meal for investigation. The result indicated that the sucrose liquid was immediately acquired into the midgut rather than the crops after a meal of the sucrose-medium mixture. The experiment was reproduced twice.

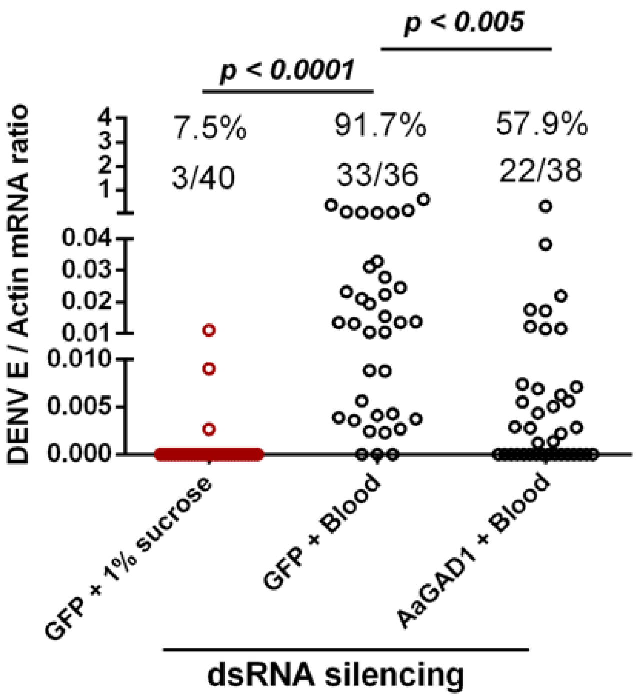

Supplementary Figure 15. Silencing *AaGAD1* offset the increase in DENV-2 infectious ratios caused by blood meals

The mosquitoes were inoculated with dsRNAs against the *AaGAD1* and *GFP* genes. Three days post-dsRNA inoculation, a mixture that containing either human blood or 1% sucrose (500 µl) and supernatant from DENV-2-infected Vero cells (cultured in serum-free medium) (500 µl) was used to feed *A. aegypti* via an *in vitro* blood feeding system. Mosquito infectivity was determined by TaqMan qPCR 8 days post-blood meal. The experiment was reproduced twice. The number of infected mosquitoes relative to the total number of mosquitoes is shown at the top of each column. Each dot represents a mosquito. The data upper mosquito number are represented as the percentage of mosquito infection. Differences in the mosquito infective ratios were compared using Fisher's exact test.

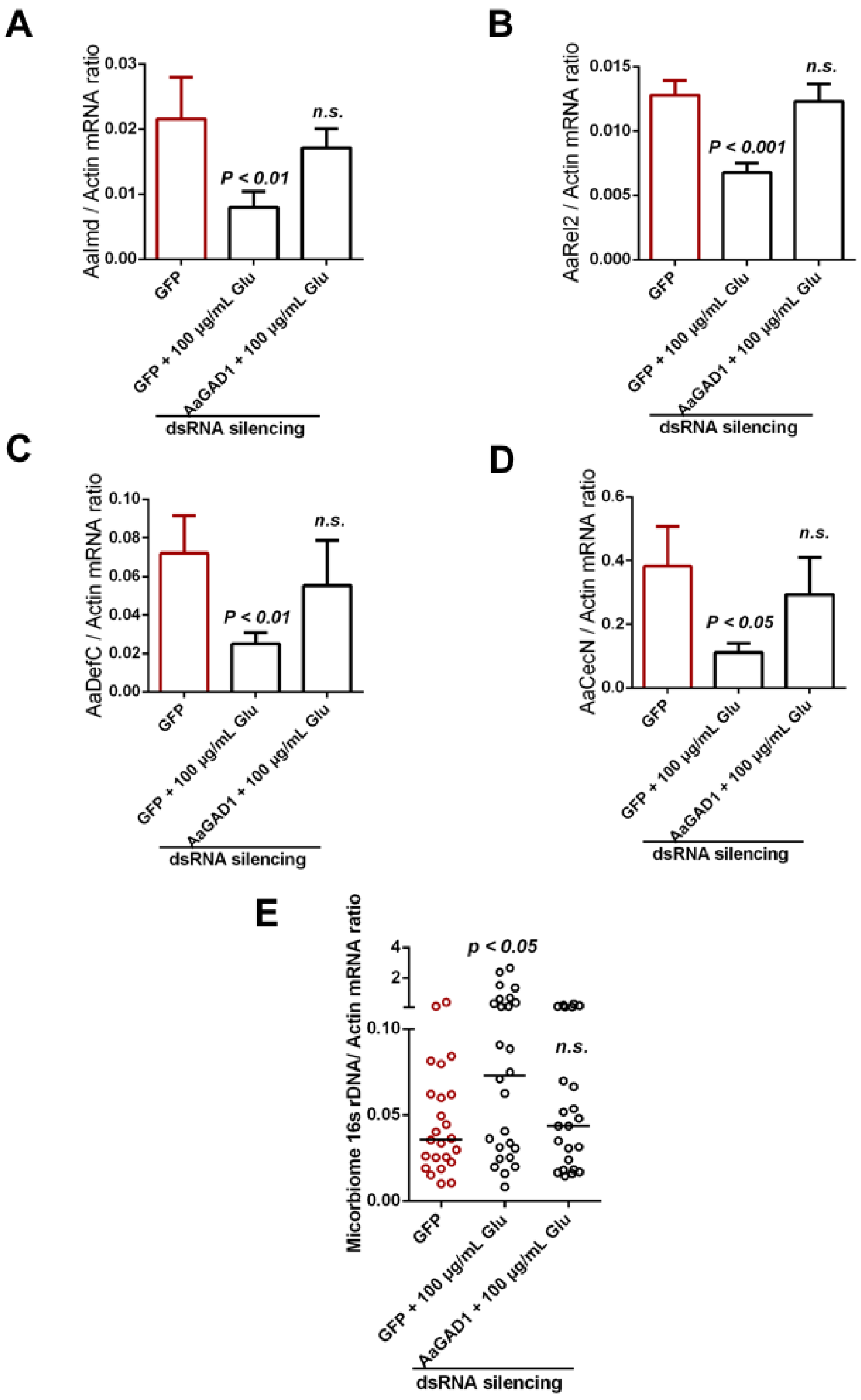

Supplementary Figure 16. Oral introduction of glutamic acid regulated the Imd pathway through the GABAergic system

(A-D) Regulation of Imd signaling by feeding with glutamic acid. Antibiotic-treated mosquitoes were inoculated with dsRNAs against the *AaGAD1* and *GFP* genes. Three days

post-dsRNA inoculation, the mosquitoes were fed a sucrose meal with 100 μg/ml glutamic acid. The antibiotic-treated mosquitoes fed with a sucrose meal without glutamic acid served as negative controls. The midguts were dissected at 18 hours after feeding to determine the expression of the *AaImd* (A), *AaRel2* (B), *AaDef-C* (C) and *AaCec-N* (D) genes by SYBR Green qPCR. The experiment was reproduced twice. The data are presented as the mean ± s.e.m.

(E) Regulation of the gut microbiome by feeding with glutamic acid. The mosquitoes, inoculated with dsRNAs against the *AaGAD1* and *GFP* genes, were orally treated with or without 100 μg/ml glutamic acid with a sucrose meal. The midguts were dissected at 24 hours after feeding. The burden of the gut bacteria was determined by qPCR. One dot represents one mosquito gut. The horizontal line represents the median value of the results.

(A-E) The data were analyzed statistically using the non-parametric *Mann-Whitney* test. The qPCR primers for the *AaImd*, *AaRel2*, *AaDef-C* and *AaCec-N* genes and the gut bacterial 16S rDNA are described in Supplementary Data 2.

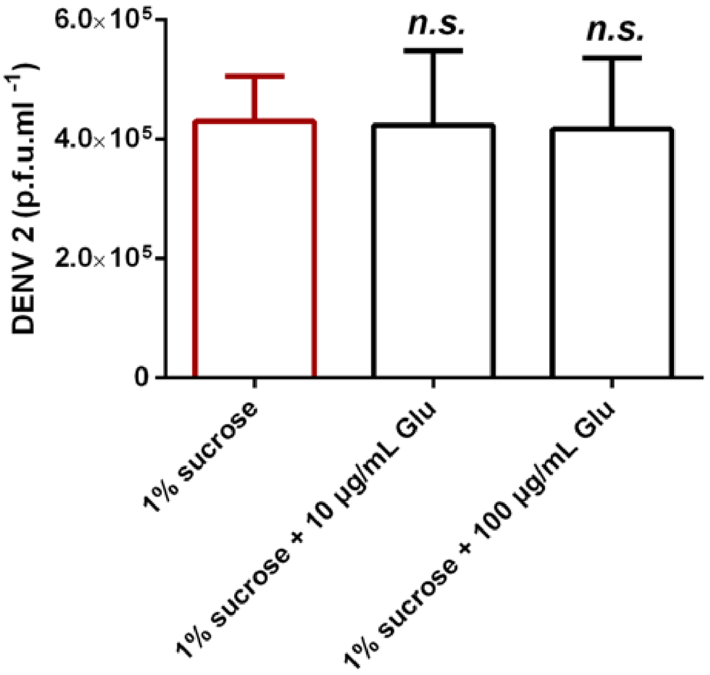

Supplementary Figure 17. The presence of glutamic acid in sucrose buffer did not affect DENV-2 infectivity

The feeding mixture, which contained 1% sucrose (50% v/v), supernatant from DENV-2-infected Vero cells (cultured in serum-free medium) (50% v/v), and 10 or 100 µg/ml glutamic acid, was maintained for 30 mins at room temperature. DENV-2 infectivity was subsequently determined by a plaque assay. The experiment was reproduced three times. The data are presented as the mean ± s.e.m.

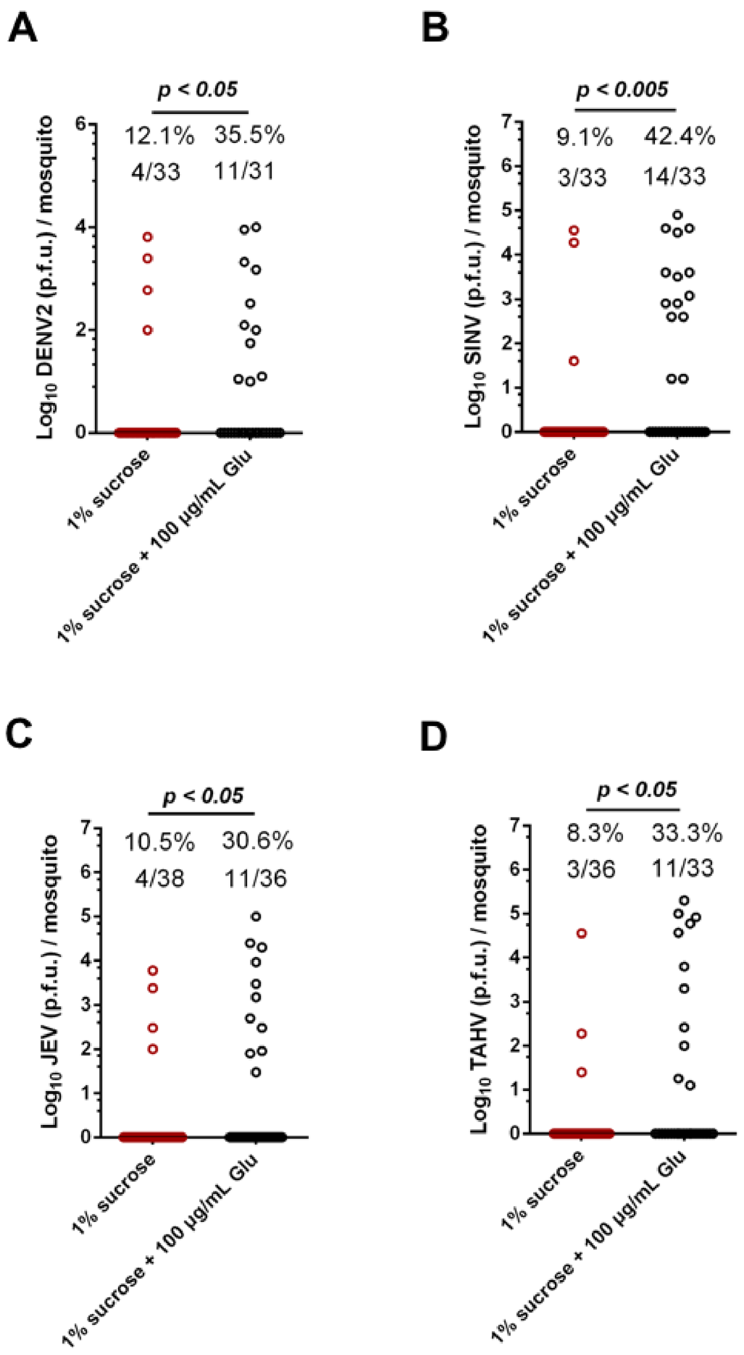

Supplementary Figure 18. Oral introduction of glutamic acid enhanced the prevalence of arbovirus infection in *A. aegypti* and *C. pipiens pallens*, as measured by plaque assay. A mixture, which contained 1% sucrose (500 µl), supernatant from virus-infected Vero cells (cultured in serum-free medium) (500 µl), and 100 µg/ml glutamic acid, was used to feed mosquitoes via an *in vitro* blood feeding system. Mosquitoes fed this mixture without glutamic acid served as a negative control. The subfigures represent the infection of DENV-2 (A) and SINV (B) in *A. aegypti*, JEV (C) and TAHV (D) in *C. pipiens pallens*. Mosquito

infectivity was determined by a plaque assays on 8 days post-sucrose meal. The experiment was reproduced twice. The number of infected mosquitoes relative to the total number of mosquitoes is shown at the top of each column. Each dot represents one mosquito. The data upper mosquito number are represented as the percentage of mosquito infection. Differences in the mosquito infective ratio were compared using Fisher's exact test.

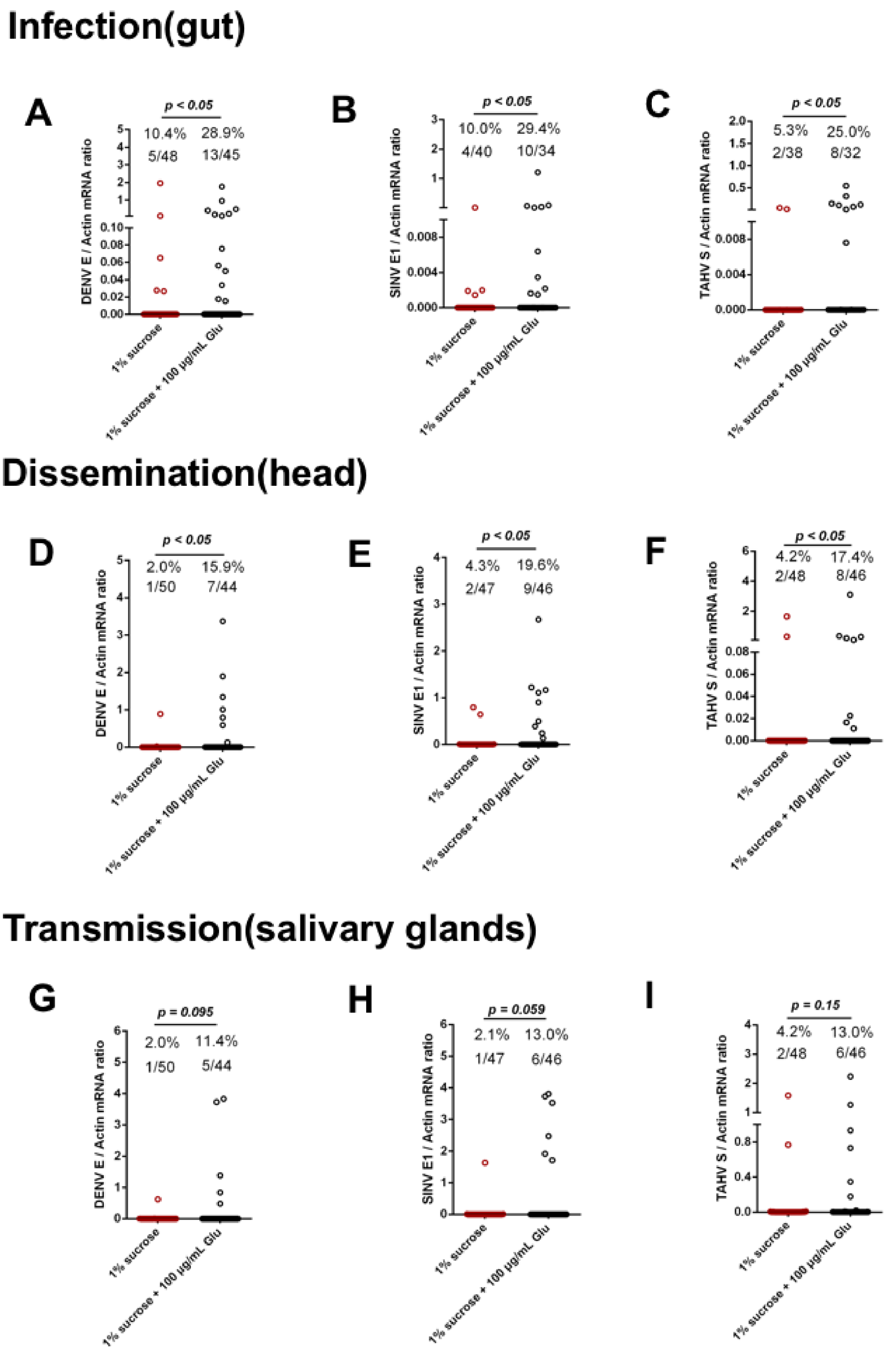

Supplementary Figure 19. The effect of the oral introduction of glutamic acid on vector competence for arbovirus infection

A mixture, which contained 1% sucrose (50% v/v), supernatant from virus-infected Vero cells (cultured in serum-free medium) (50% v/v), and 100 µg/ml glutamic acid, was used to feed

mosquitoes via an *in vitro* blood feeding system. Mosquitoes feeding on the mixture without glutamic acid served as negative controls. The midguts, heads and salivary glands were dissected on 7, 14 and 14 days after oral feeding, respectively. The viral loads were measured by qPCR. The ratio of midgut infection, head dissemination and salivary glands infection (transmission) were calculated by the Positive Number / the Total Number in the midguts (A-C), heads (D-F) and salivary glands (G-I), respectively. The experiment was reproduced twice. The number of infected mosquitoes relative to the total number of mosquitoes is shown at the top of each column. Each dot represents one mosquito. The data upper mosquito number are represented as the percentage of mosquito infection. Differences in the mosquito infective ratio were compared using Fisher's exact test.

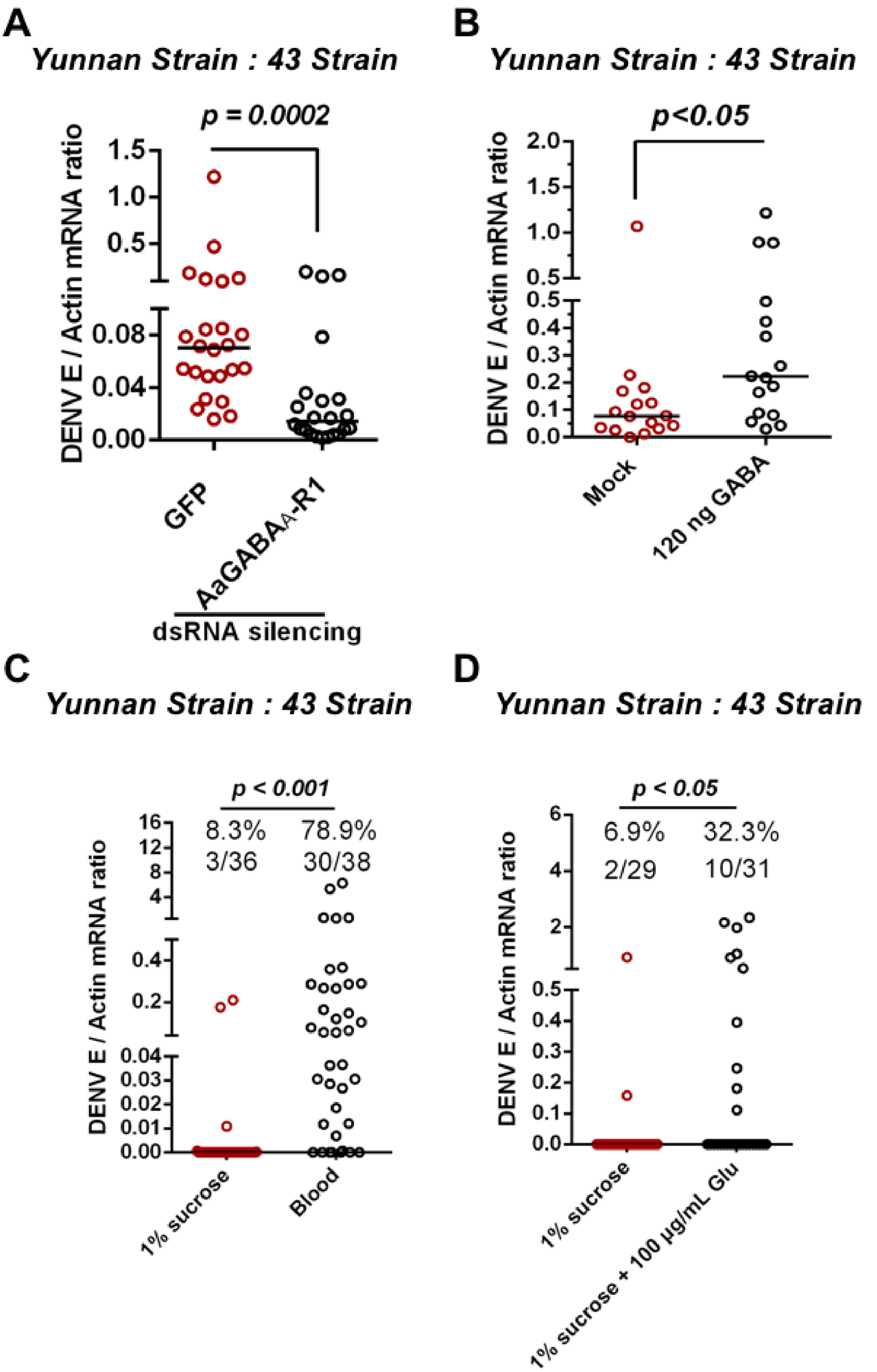

Supplementary Figure 20. The role of the GABAergic system in the infection of a low-passage DENV strain in a field *A. aegypti* strain

(A) Knockdown of *AaGABA*$_A$*-R1* impaired the infection of the DENV-2 43 strain in the *A. aegypti* Yunnan mosquitoes.

(B) Thoracic inoculation of GABA enhanced the DENV-2 43 infection in the *A. aegypti* Yunnan mosquitoes.

(C) Feeding blood enhanced the infection of DENV-2 43 strain in the *A. aegypti* Yunnan mosquitoes. Either human blood or 1% sucrose (500 µl) were premixed with supernatant from DENV-2-infected Vero cells (cultured in serum-free medium) (500 µl) was used to feed mosquitoes via an *in vitro* membrane blood meal.

(D) Oral introduction of glutamic acid enhanced the prevalence of DENV-2 43 strain infection in the *A. aegypti* Yunnan mosquitoes. A mixture, which contained 1% sucrose (500 µl), supernatant from DENV-2-infected Vero cells (cultured in serum-free medium) (500 µl), and 100 µg/ml glutamic acid, was used to feed *A. aegypti* via an *in vitro* blood feeding system. Mosquitoes fed the mixture without glutamic acid served as negative controls.

(A-B) The viral loads were assessed at 3 days post-infection via TaqMan qPCR and were normalized to *A. aegypti actin* (*AAEL011197*). One dot represents 1 mosquito, and the horizontal line represents the median of the results. The data were analyzed statistically using the non-parametric Mann-Whitney test.

(C-D) Mosquito infectivity was determined by TaqMan qPCR 8 days post-blood meal. The number of infected mosquitoes relative to the total number of mosquitoes is shown at the top of each column. Each dot represents one mosquito. The data upper mosquito number are represented as the percentage of mosquito infection. Differences in the mosquito infective ratio were compared using Fisher's exact test.

(A-D) The primers and probes used for PCR are presented in Supplementary Data 2. The results from at least 2 independent experiments were combined.
