# Supplementary material for: Blood meal acquisition enhances arbovirus replication in mosquitoes through activation of the GABAergic system
Source: Nat Commun. 2017 Nov 2;8:1262. doi: 10.1038/s41467-017-01244-6 (PMC5665997; doi:10.1038/s41467-017-01244-6)
Supplement: Supplementary file 3 — Description of Additional Supplementary Files [file 41467_2017_1244_MOESM3_ESM.pdf]

File name: Supplementary Data 1
Description: Regulation of immune gene in the mosquitoes after silencing the *AaGABA*$_A$*-R1*

File name: Supplementary Data 2
Description: Primers and probe for qPCR, dsRNA synthesis
